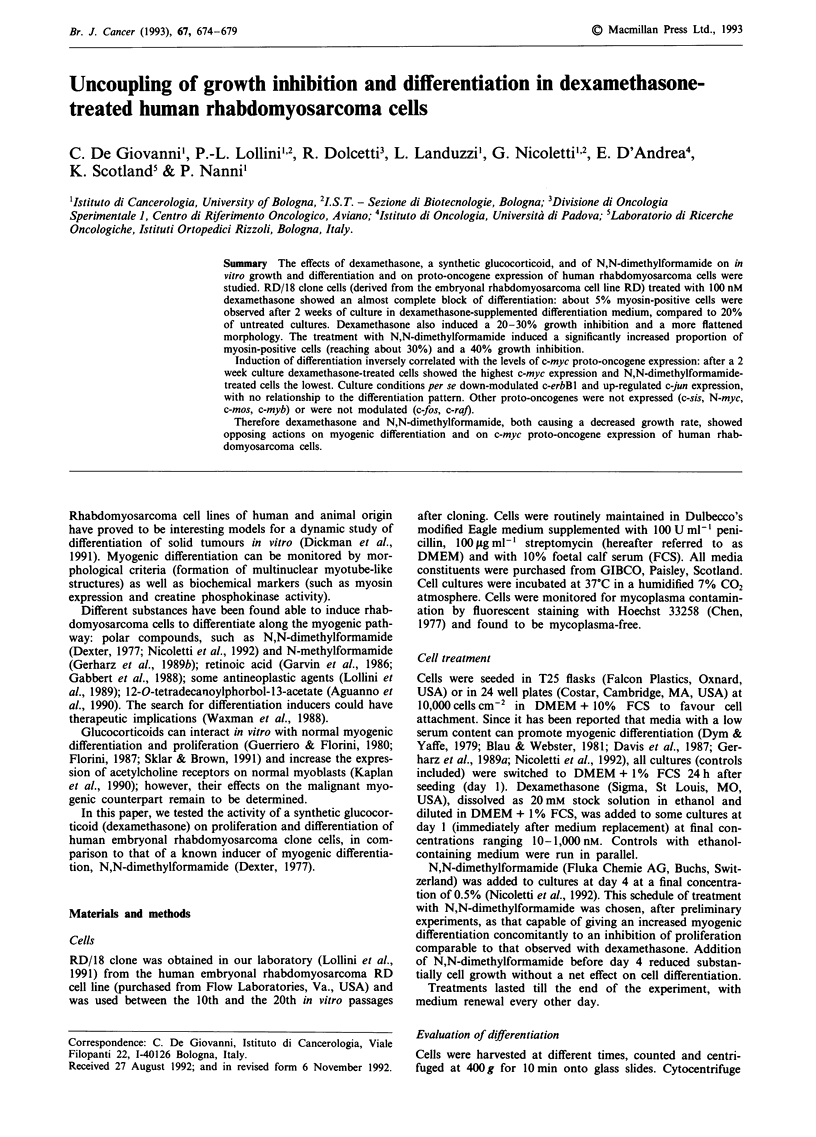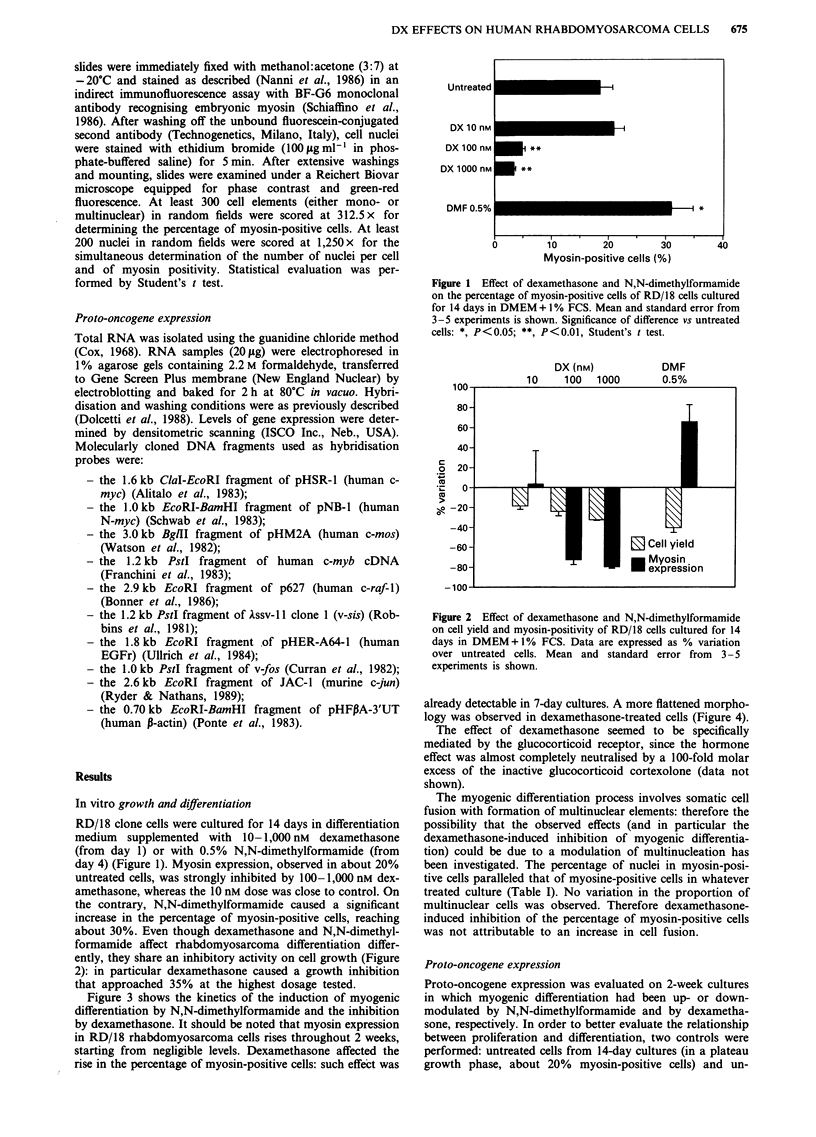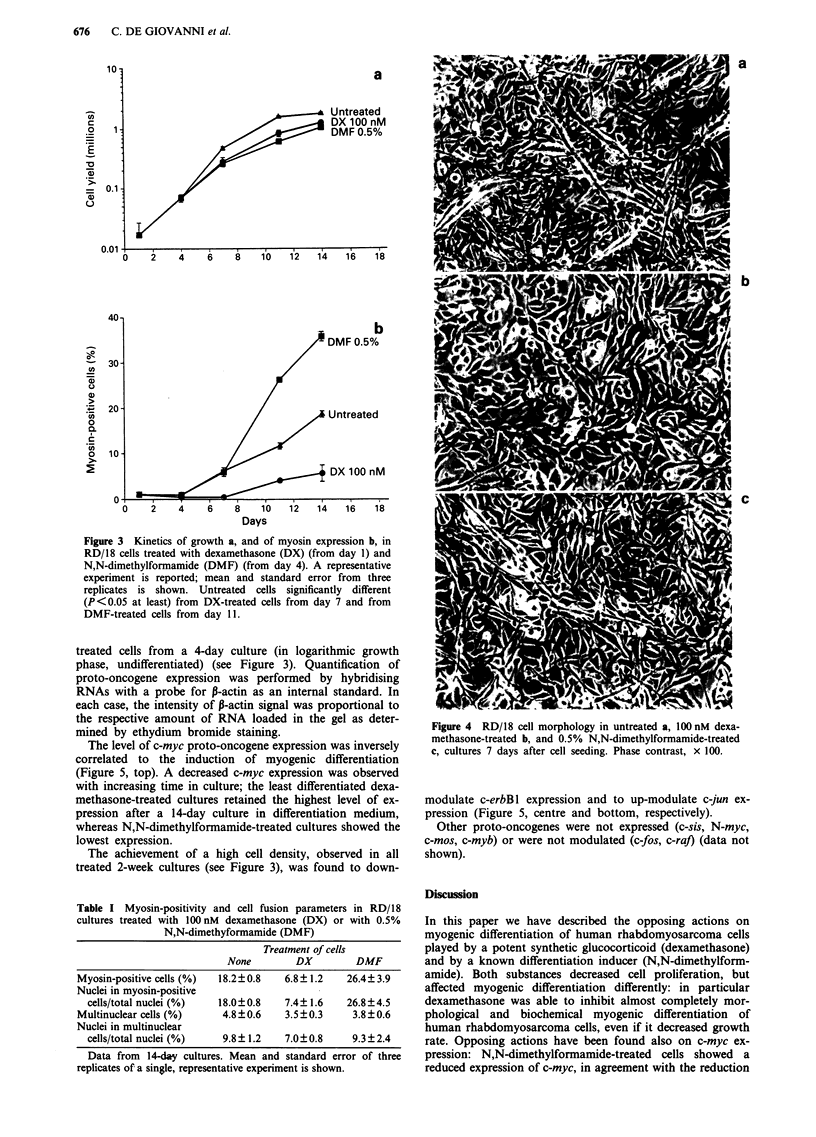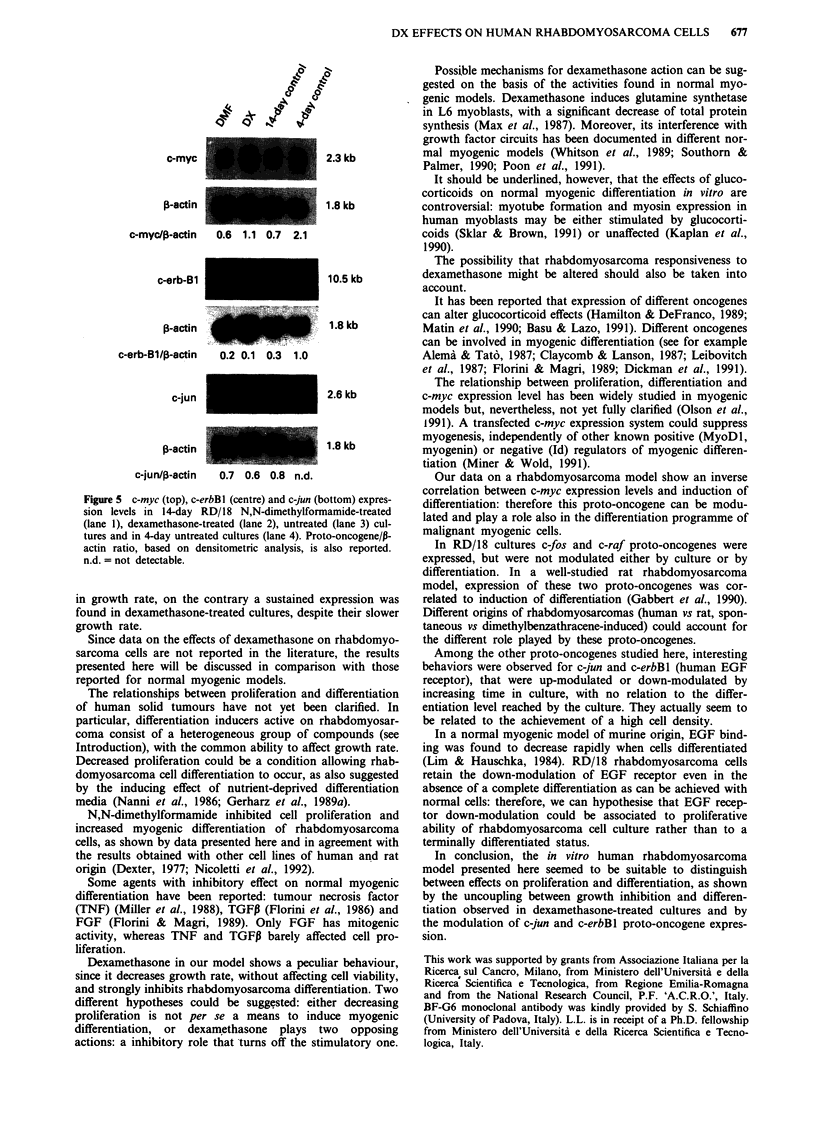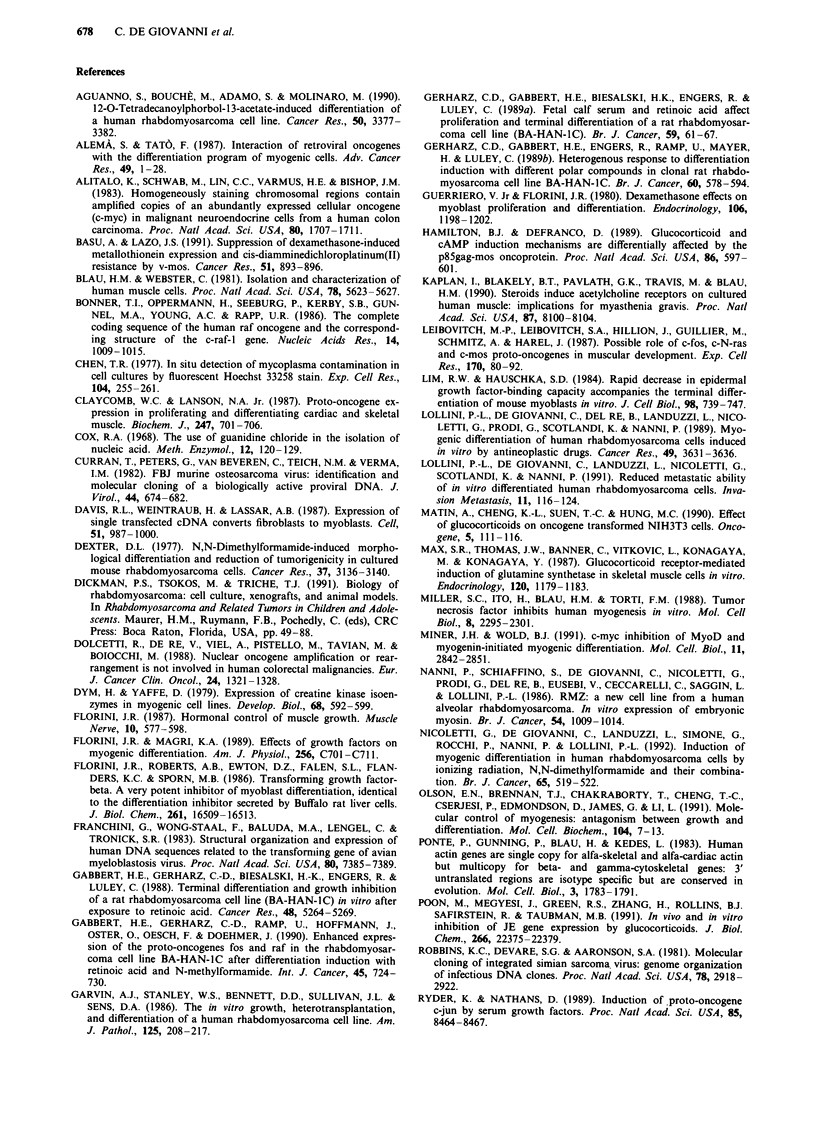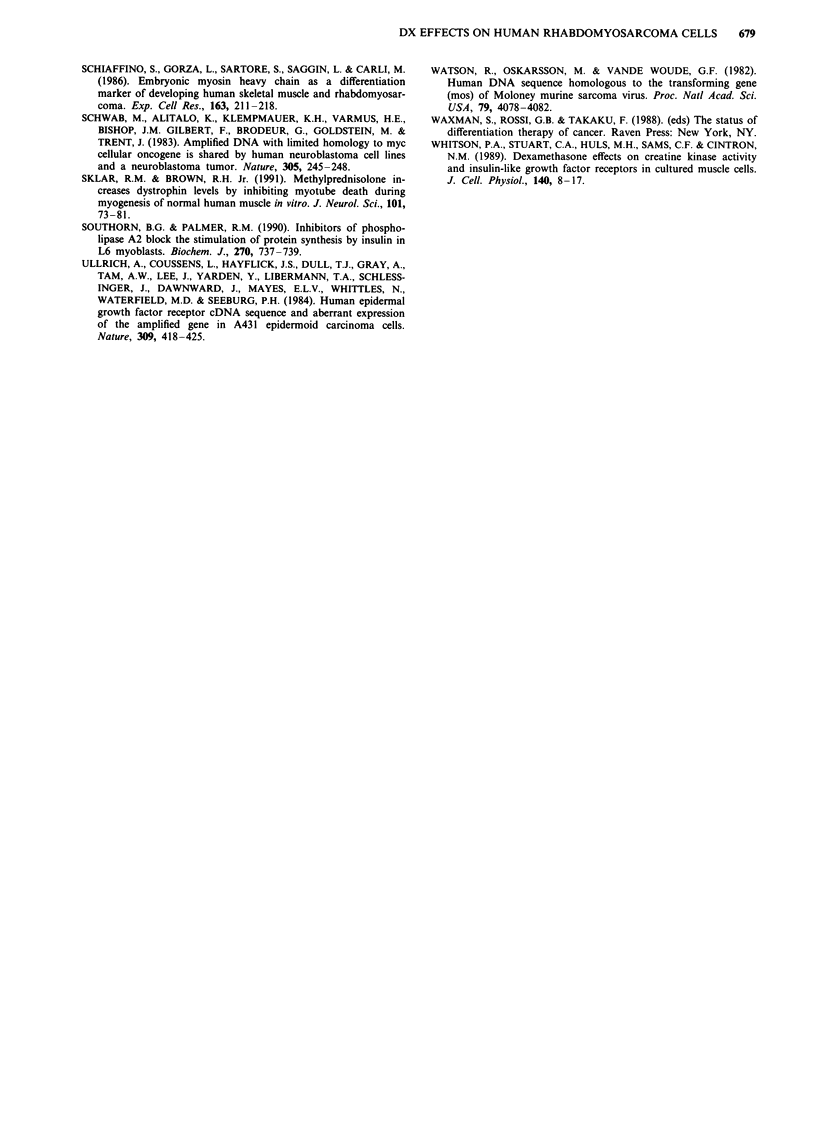# Uncoupling of growth inhibition and differentiation in dexamethasone-treated human rhabdomyosarcoma cells.

**DOI:** 10.1038/bjc.1993.125

**Published:** 1993-04

**Authors:** C. De Giovanni, P. L. Lollini, R. Dolcetti, L. Landuzzi, G. Nicoletti, E. D'Andrea, K. Scotland, P. Nanni

**Affiliations:** Istituto di Cancerologia, University of Bologna, Italy.

## Abstract

**Images:**


Br. J. Cancer (1993), 67, 674–679 © Macmillan Press Ltd., 1993

### Uncoupling of growth inhibition and differentiation in dexamethasone-treated human rhabdomyosarcoma cells

C. De Giovanni[1], P.-L. Lollini[1,2], R. Dolcetti[3], L. Landuzzi[1], G. Nicoletti[1,2], E. D'Andrea[4], K. Scotland[5] & P. Nanni[1]

[1]*Istituto di Cancerologia, University of Bologna,* [2]*I.S.T. – Sezione di Biotecnologie, Bologna;* [3]*Divisione di Oncologia Sperimentale 1, Centro di Riferimento Oncologico, Aviano;* [4]*Istituto di Oncologia, Università di Padova;* [5]*Laboratorio di Ricerche Oncologiche, Istituti Ortopedici Rizzoli, Bologna, Italy.*

**Summary** The effects of dexamethasone, a synthetic glucocorticoid, and of N,N-dimethylformamide on *in vitro* growth and differentiation and on proto-oncogene expression of human rhabdomyosarcoma cells were studied. RD/18 clone cells (derived from the embryonal rhabdomyosarcoma cell line RD) treated with 100 nM dexamethasone showed an almost complete block of differentiation: about 5% myosin-positive cells were observed after 2 weeks of culture in dexamethasone-supplemented differentiation medium, compared to 20% of untreated cultures. Dexamethasone also induced a 20–30% growth inhibition and a more flattened morphology. The treatment with N,N-dimethylformamide induced a significantly increased proportion of myosin-positive cells (reaching about 30%) and a 40% growth inhibition.

Induction of differentiation inversely correlated with the levels of c-*myc* proto-oncogene expression: after a 2 week culture dexamethasone-treated cells showed the highest c-*myc* expression and N,N-dimethylformamide-treated cells the lowest. Culture conditions *per se* down-modulated c-*erb*B1 and up-regulated c-*jun* expression, with no relationship to the differentiation pattern. Other proto-oncogenes were not expressed (c-*sis*, N-*myc*, c-*mos*, c-*myb*) or were not modulated (c-*fos*, c-*raf*).

Therefore dexamethasone and N,N-dimethylformamide, both causing a decreased growth rate, showed opposing actions on myogenic differentiation and on c-*myc* proto-oncogene expression of human rhabdomyosarcoma cells.

Rhabdomyosarcoma cell lines of human and animal origin have proved to be interesting models for a dynamic study of differentiation of solid tumours *in vitro* (Dickman *et al.*, 1991). Myogenic differentiation can be monitored by morphological criteria (formation of multinuclear myotube-like structures) as well as biochemical markers (such as myosin expression and creatine phosphokinase activity).

Different substances have been found able to induce rhabdomyosarcoma cells to differentiate along the myogenic pathway: polar compounds, such as N,N-dimethylformamide (Dexter, 1977; Nicoletti *et al.*, 1992) and N-methylformamide (Gerharz *et al.*, 1989*b*); retinoic acid (Garvin *et al.*, 1986; Gabbert *et al.*, 1988); some antineoplastic agents (Lollini *et al.*, 1989); 12-*O*-tetradecanoylphorbol-13-acetate (Aguanno *et al.*, 1990). The search for differentiation inducers could have therapeutic implications (Waxman *et al.*, 1988).

Glucocorticoids can interact *in vitro* with normal myogenic differentiation and proliferation (Guerriero & Florini, 1980; Florini, 1987; Sklar & Brown, 1991) and increase the expression of acetylcholine receptors on normal myoblasts (Kaplan *et al.*, 1990); however, their effects on the malignant myogenic counterpart remain to be determined.

In this paper, we tested the activity of a synthetic glucocorticoid (dexamethasone) on proliferation and differentiation of human embryonal rhabdomyosarcoma clone cells, in comparison to that of a known inducer of myogenic differentiation, N,N-dimethylformamide (Dexter, 1977).

### Materials and methods

### *Cells*

RD/18 clone was obtained in our laboratory (Lollini *et al.*, 1991) from the human embryonal rhabdomyosarcoma RD cell line (purchased from Flow Laboratories, Va., USA) and was used between the 10th and the 20th *in vitro* passages after cloning. Cells were routinely maintained in Dulbecco's modified Eagle medium supplemented with 100 U $ml^{-1}$ penicillin, 100 $\mu g\, ml^{-1}$ streptomycin (hereafter referred to as DMEM) and with 10% foetal calf serum (FCS). All media constituents were purchased from GIBCO, Paisley, Scotland. Cell cultures were incubated at 37°C in a humidified 7% $CO_2$ atmosphere. Cells were monitored for mycoplasma contamination by fluorescent staining with Hoechst 33258 (Chen, 1977) and found to be mycoplasma-free.

Correspondence: C. De Giovanni, Istituto di Cancerologia, Viale Filopanti 22, I-40126 Bologna, Italy.
Received 27 August 1992; and in revised form 6 November 1992.

### *Cell treatment*

Cells were seeded in T25 flasks (Falcon Plastics, Oxnard, USA) or in 24 well plates (Costar, Cambridge, MA, USA) at 10,000 cells $cm^{-2}$ in DMEM + 10% FCS to favour cell attachment. Since it has been reported that media with a low serum content can promote myogenic differentiation (Dym & Yaffe, 1979; Blau & Webster, 1981; Davis *et al.*, 1987; Gerharz *et al.*, 1989*a*; Nicoletti *et al.*, 1992), all cultures (controls included) were switched to DMEM + 1% FCS 24 h after seeding (day 1). Dexamethasone (Sigma, St Louis, MO, USA), dissolved as 20 mM stock solution in ethanol and diluted in DMEM + 1% FCS, was added to some cultures at day 1 (immediately after medium replacement) at final concentrations ranging 10–1,000 nM. Controls with ethanol-containing medium were run in parallel.

N,N-dimethylformamide (Fluka Chemie AG, Buchs, Switzerland) was added to cultures at day 4 at a final concentration of 0.5% (Nicoletti *et al.*, 1992). This schedule of treatment with N,N-dimethylformamide was chosen, after preliminary experiments, as that capable of giving an increased myogenic differentiation concomitantly to an inhibition of proliferation comparable to that observed with dexamethasone. Addition of N,N-dimethylformamide before day 4 reduced substantially cell growth without a net effect on cell differentiation.

Treatments lasted till the end of the experiment, with medium renewal every other day.

### *Evaluation of differentiation*

Cells were harvested at different times, counted and centrifuged at 400 *g* for 10 min onto glass slides. Cytocentrifuge slides were immediately fixed with methanol:acetone (3:7) at −20°C and stained as described (Nanni *et al.*, 1986) in an indirect immunofluorescence assay with BF-G6 monoclonal antibody recognising embryonic myosin (Schiaffino *et al.*, 1986). After washing off the unbound fluorescein-conjugated second antibody (Technogenetics, Milano, Italy), cell nuclei were stained with ethidium bromide (100 $\mu g\ ml^{-1}$ in phosphate-buffered saline) for 5 min. After extensive washings and mounting, slides were examined under a Reichert Biovar microscope equipped for phase contrast and green-red fluorescence. At least 300 cell elements (either mono- or multinuclear) in random fields were scored at 312.5× for determining the percentage of myosin-positive cells. At least 200 nuclei in random fields were scored at 1,250× for the simultaneous determination of the number of nuclei per cell and of myosin positivity. Statistical evaluation was performed by Student's *t* test.

### *Proto-oncogene expression*

Total RNA was isolated using the guanidine chloride method (Cox, 1968). RNA samples (20 μg) were electrophoresed in 1% agarose gels containing 2.2 M formaldehyde, transferred to Gene Screen Plus membrane (New England Nuclear) by electroblotting and baked for 2 h at 80°C *in vacuo*. Hybridisation and washing conditions were as previously described (Dolcetti *et al.*, 1988). Levels of gene expression were determined by densitometric scanning (ISCO Inc., Neb., USA). Molecularly cloned DNA fragments used as hybridisation probes were:

- the 1.6 kb *ClaI-EcoRI* fragment of pHSR-1 (human c-*myc*) (Alitalo *et al.*, 1983);
- the 1.0 kb *EcoRI-BamHI* fragment of pNB-1 (human N-*myc*) (Schwab *et al.*, 1983);
- the 3.0 kb *BglII* fragment of pHM2A (human c-*mos*) (Watson *et al.*, 1982);
- the 1.2 kb *PstI* fragment of human c-*myb* cDNA (Franchini *et al.*, 1983);
- the 2.9 kb *EcoRI* fragment of p627 (human c-*raf*-1) (Bonner *et al.*, 1986);
- the 1.2 kb *PstI* fragment of λssv-11 clone 1 (v-*sis*) (Robbins *et al.*, 1981);
- the 1.8 kb *EcoRI* fragment of pHER-A64-1 (human EGFr) (Ullrich *et al.*, 1984);
- the 1.0 kb *PstI* fragment of v-*fos* (Curran *et al.*, 1982);
- the 2.6 kb *EcoRI* fragment of JAC-1 (murine c-*jun*) (Ryder & Nathans, 1989);
- the 0.70 kb *EcoRI-BamHI* fragment of pHFβA-3'UT (human β-actin) (Ponte *et al.*, 1983).

### Results

### *In vitro growth and differentiation*

RD/18 clone cells were cultured for 14 days in differentiation medium supplemented with 10–1,000 nM dexamethasone (from day 1) or with 0.5% N,N-dimethylformamide (from day 4) (Figure 1). Myosin expression, observed in about 20% untreated cells, was strongly inhibited by 100–1,000 nM dexamethasone, whereas the 10 nM dose was close to control. On the contrary, N,N-dimethylformamide caused a significant increase in the percentage of myosin-positive cells, reaching about 30%. Even though dexamethasone and N,N-dimethylformamide affect rhabdomyosarcoma differentiation differently, they share an inhibitory activity on cell growth (Figure 2): in particular dexamethasone caused a growth inhibition that approached 35% at the highest dosage tested.

Figure 3 shows the kinetics of the induction of myogenic differentiation by N,N-dimethylformamide and the inhibition by dexamethasone. It should be noted that myosin expression in RD/18 rhabdomyosarcoma cells rises throughout 2 weeks, starting from negligible levels. Dexamethasone affected the rise in the percentage of myosin-positive cells: such effect was

**Figure 1** Effect of dexamethasone and N,N-dimethylformamide on the percentage of myosin-positive cells of RD/18 cells cultured for 14 days in DMEM + 1% FCS. Mean and standard error from 3–5 experiments is shown. Significance of difference *vs* untreated cells: *, $P<0.05$; **, $P<0.01$, Student's *t* test.

**Figure 2** Effect of dexamethasone and N,N-dimethylformamide on cell yield and myosin-positivity of RD/18 cells cultured for 14 days in DMEM + 1% FCS. Data are expressed as % variation over untreated cells. Mean and standard error from 3–5 experiments is shown.

already detectable in 7-day cultures. A more flattened morphology was observed in dexamethasone-treated cells (Figure 4).

The effect of dexamethasone seemed to be specifically mediated by the glucocorticoid receptor, since the hormone effect was almost completely neutralised by a 100-fold molar excess of the inactive glucocorticoid cortexolone (data not shown).

The myogenic differentiation process involves somatic cell fusion with formation of multinuclear elements: therefore the possibility that the observed effects (and in particular the dexamethasone-induced inhibition of myogenic differentiation) could be due to a modulation of multinucleation has been investigated. The percentage of nuclei in myosin-positive cells paralleled that of myosine-positive cells in whatever treated culture (Table I). No variation in the proportion of multinuclear cells was observed. Therefore dexamethasone-induced inhibition of the percentage of myosin-positive cells was not attributable to an increase in cell fusion.

### *Proto-oncogene expression*

Proto-oncogene expression was evaluated on 2-week cultures in which myogenic differentiation had been up- or down-modulated by N,N-dimethylformamide and by dexamethasone, respectively. In order to better evaluate the relationship between proliferation and differentiation, two controls were performed: untreated cells from 14-day cultures (in a plateau growth phase, about 20% myosin-positive cells) and un-

**Figure 3** Kinetics of growth **a**, and of myosin expression **b**, in RD/18 cells treated with dexamethasone (DX) (from day 1) and N,N-dimethylformamide (DMF) (from day 4). A representative experiment is reported; mean and standard error from three replicates is shown. Untreated cells significantly different ($P<0.05$ at least) from DX-treated cells from day 7 and from DMF-treated cells from day 11.

treated cells from a 4-day culture (in logarithmic growth phase, undifferentiated) (see Figure 3). Quantification of proto-oncogene expression was performed by hybridising RNAs with a probe for β-actin as an internal standard. In each case, the intensity of β-actin signal was proportional to the respective amount of RNA loaded in the gel as determined by ethydium bromide staining.

The level of c-*myc* proto-oncogene expression was inversely correlated to the induction of myogenic differentiation (Figure 5, top). A decreased c-*myc* expression was observed with increasing time in culture; the least differentiated dexamethasone-treated cultures retained the highest level of expression after a 14-day culture in differentiation medium, whereas N,N-dimethylformamide-treated cultures showed the lowest expression.

The achievement of a high cell density, observed in all treated 2-week cultures (see Figure 3), was found to down-

**Table I** Myosin-positivity and cell fusion parameters in RD/18 cultures treated with 100 nM dexamethasone (DX) or with 0.5% N,N-dimethyformamide (DMF)

| | *Treatment of cells* | | |
|---|---|---|---|
| | *None* | *DX* | *DMF* |
| Myosin-positive cells (%) | 18.2±0.8 | 6.8±1.2 | 26.4±3.9 |
| Nuclei in myosin-positive cells/total nuclei (%) | 18.0±0.8 | 7.4±1.6 | 26.8±4.5 |
| Multinuclear cells (%) | 4.8±0.6 | 3.5±0.3 | 3.8±0.6 |
| Nuclei in multinuclear cells/total nuclei (%) | 9.8±1.2 | 7.0±0.8 | 9.3±2.4 |

Data from 14-day cultures. Mean and standard error of three replicates of a single, representative experiment is shown.

**Figure 4** RD/18 cell morphology in untreated **a**, 100 nM dexamethasone-treated **b**, and 0.5% N,N-dimethylformamide-treated **c**, cultures 7 days after cell seeding. Phase contrast, × 100.

modulate c-*erb*B1 expression and to up-modulate c-*jun* expression (Figure 5, centre and bottom, respectively).

Other proto-oncogenes were not expressed (c-*sis*, N-*myc*, c-*mos*, c-*myb*) or were not modulated (c-*fos*, c-*raf*) (data not shown).

### Discussion

In this paper we have described the opposing actions on myogenic differentiation of human rhabdomyosarcoma cells played by a potent synthetic glucocorticoid (dexamethasone) and by a known differentiation inducer (N,N-dimethylformamide). Both substances decreased cell proliferation, but affected myogenic differentiation differently: in particular dexamethasone was able to inhibit almost completely morphological and biochemical myogenic differentiation of human rhabdomyosarcoma cells, even if it decreased growth rate. Opposing actions have been found also on c-*myc* expression: N,N-dimethylformamide-treated cells showed a reduced expression of c-*myc*, in agreement with the reduction

**Figure 5** c-*myc* (top), c-*erb*B1 (centre) and c-*jun* (bottom) expression levels in 14-day RD/18 N,N-dimethylformamide-treated (lane 1), dexamethasone-treated (lane 2), untreated (lane 3) cultures and in 4-day untreated cultures (lane 4). Proto-oncogene/β-actin ratio, based on densitometric analysis, is also reported. n.d. = not detectable.

in growth rate, on the contrary a sustained expression was found in dexamethasone-treated cultures, despite their slower growth rate.

Since data on the effects of dexamethasone on rhabdomyosarcoma cells are not reported in the literature, the results presented here will be discussed in comparison with those reported for normal myogenic models.

The relationships between proliferation and differentiation of human solid tumours have not yet been clarified. In particular, differentiation inducers active on rhabdomyosarcoma consist of a heterogeneous group of compounds (see Introduction), with the common ability to affect growth rate. Decreased proliferation could be a condition allowing rhabdomyosarcoma cell differentiation to occur, as also suggested by the inducing effect of nutrient-deprived differentiation media (Nanni *et al.*, 1986; Gerharz *et al.*, 1989*a*).

N,N-dimethylformamide inhibited cell proliferation and increased myogenic differentiation of rhabdomyosarcoma cells, as shown by data presented here and in agreement with the results obtained with other cell lines of human and rat origin (Dexter, 1977; Nicoletti *et al.*, 1992).

Some agents with inhibitory effect on normal myogenic differentiation have been reported: tumour necrosis factor (TNF) (Miller *et al.*, 1988), TGFβ (Florini *et al.*, 1986) and FGF (Florini & Magri, 1989). Only FGF has mitogenic activity, whereas TNF and TGFβ barely affected cell proliferation.

Dexamethasone in our model shows a peculiar behaviour, since it decreases growth rate, without affecting cell viability, and strongly inhibits rhabdomyosarcoma differentiation. Two different hypotheses could be suggested: either decreasing proliferation is not *per se* a means to induce myogenic differentiation, or dexamethasone plays two opposing actions: a inhibitory role that turns off the stimulatory one.

Possible mechanisms for dexamethasone action can be suggested on the basis of the activities found in normal myogenic models. Dexamethasone induces glutamine synthetase in L6 myoblasts, with a significant decrease of total protein synthesis (Max *et al.*, 1987). Moreover, its interference with growth factor circuits has been documented in different normal myogenic models (Whitson *et al.*, 1989; Southorn & Palmer, 1990; Poon *et al.*, 1991).

It should be underlined, however, that the effects of glucocorticoids on normal myogenic differentiation *in vitro* are controversial: myotube formation and myosin expression in human myoblasts may be either stimulated by glucocorticoids (Sklar & Brown, 1991) or unaffected (Kaplan *et al.*, 1990).

The possibility that rhabdomyosarcoma responsiveness to dexamethasone might be altered should also be taken into account.

It has been reported that expression of different oncogenes can alter glucocorticoid effects (Hamilton & DeFranco, 1989; Matin *et al.*, 1990; Basu & Lazo, 1991). Different oncogenes can be involved in myogenic differentiation (see for example Alemà & Tatò, 1987; Claycomb & Lanson, 1987; Leibovitch *et al.*, 1987; Florini & Magri, 1989; Dickman *et al.*, 1991).

The relationship between proliferation, differentiation and c-*myc* expression level has been widely studied in myogenic models but, nevertheless, not yet fully clarified (Olson *et al.*, 1991). A transfected c-*myc* expression system could suppress myogenesis, independently of other known positive (MyoD1, myogenin) or negative (Id) regulators of myogenic differentiation (Miner & Wold, 1991).

Our data on a rhabdomyosarcoma model show an inverse correlation between c-*myc* expression levels and induction of differentiation: therefore this proto-oncogene can be modulated and play a role also in the differentiation programme of malignant myogenic cells.

In RD/18 cultures c-*fos* and c-*raf* proto-oncogenes were expressed, but were not modulated either by culture or by differentiation. In a well-studied rat rhabdomyosarcoma model, expression of these two proto-oncogenes was correlated to induction of differentiation (Gabbert *et al.*, 1990). Different origins of rhabdomyosarcomas (human *vs* rat, spontaneous *vs* dimethylbenzathracene-induced) could account for the different role played by these proto-oncogenes.

Among the other proto-oncogenes studied here, interesting behaviors were observed for c-*jun* and c-*erb*B1 (human EGF receptor), that were up-modulated or down-modulated by increasing time in culture, with no relation to the differentiation level reached by the culture. They actually seem to be related to the achievement of a high cell density.

In a normal myogenic model of murine origin, EGF binding was found to decrease rapidly when cells differentiated (Lim & Hauschka, 1984). RD/18 rhabdomyosarcoma cells retain the down-modulation of EGF receptor even in the absence of a complete differentiation as can be achieved with normal cells: therefore, we can hypothesise that EGF receptor down-modulation could be associated to proliferative ability of rhabdomyosarcoma cell culture rather than to a terminally differentiated status.

In conclusion, the *in vitro* human rhabdomyosarcoma model presented here seemed to be suitable to distinguish between effects on proliferation and differentiation, as shown by the uncoupling between growth inhibition and differentiation observed in dexamethasone-treated cultures and by the modulation of c-*jun* and c-*erb*B1 proto-oncogene expression.

This work was supported by grants from Associazione Italiana per la Ricerca sul Cancro, Milano, from Ministero dell'Università e della Ricerca Scientifica e Tecnologica, from Regione Emilia-Romagna and from the National Research Council, P.F. 'A.C.R.O.', Italy. BF-G6 monoclonal antibody was kindly provided by S. Schiaffino (University of Padova, Italy). L.L. is in receipt of a Ph.D. fellowship from Ministero dell'Università e della Ricerca Scientifica e Tecnologica, Italy.

### References

AGUANNO, S., BOUCHÈ, M., ADAMO, S. & MOLINARO, M. (1990). 12-O-Tetradecanoylphorbol-13-acetate-induced differentiation of a human rhabdomyosarcoma cell line. *Cancer Res.*, **50**, 3377-3382.

ALEMÀ, S. & TATÒ, F. (1987). Interaction of retroviral oncogenes with the differentiation program of myogenic cells. *Adv. Cancer Res.*, **49**, 1-28.

ALITALO, K., SCHWAB, M., LIN, C.C., VARMUS, H.E. & BISHOP, J.M. (1983). Homogeneously staining chromosomal regions contain amplified copies of an abundantly expressed cellular oncogene (c-myc) in malignant neuroendocrine cells from a human colon carcinoma. *Proc. Natl Acad. Sci. USA*, **80**, 1707-1711.

BASU, A. & LAZO, J.S. (1991). Suppression of dexamethasone-induced metallothionein expression and cis-diamminedichloroplatinum(II) resistance by v-mos. *Cancer Res.*, **51**, 893-896.

BLAU, H.M. & WEBSTER, C. (1981). Isolation and characterization of human muscle cells. *Proc. Natl Acad. Sci. USA*, **78**, 5623-5627.

BONNER, T.I., OPPERMANN, H., SEEBURG, P., KERBY, S.B., GUNNEL, M.A., YOUNG, A.C. & RAPP, U.R. (1986). The complete coding sequence of the human raf oncogene and the corresponding structure of the c-raf-1 gene. *Nucleic Acids Res.*, **14**, 1009-1015.

CHEN, T.R. (1977). In situ detection of mycoplasma contamination in cell cultures by fluorescent Hoechst 33258 stain. *Exp. Cell Res.*, **104**, 255-261.

CLAYCOMB, W.C. & LANSON, N.A. Jr. (1987). Proto-oncogene expression in proliferating and differentiating cardiac and skeletal muscle. *Biochem. J.*, **247**, 701-706.

COX, R.A. (1968). The use of guanidine chloride in the isolation of nucleic acid. *Meth. Enzymol.*, **12**, 120-129.

CURRAN, T., PETERS, G., VAN BEVEREN, C., TEICH, N.M. & VERMA, I.M. (1982). FBJ murine osteosarcoma virus: identification and molecular cloning of a biologically active proviral DNA. *J. Virol.*, **44**, 674-682.

DAVIS, R.L., WEINTRAUB, H. & LASSAR, A.B. (1987). Expression of single transfected cDNA converts fibroblasts to myoblasts. *Cell*, **51**, 987-1000.

DEXTER, D.L. (1977). N,N-Dimethylformamide-induced morphological differentiation and reduction of tumorigenicity in cultured mouse rhabdomyosarcoma cells. *Cancer Res.*, **37**, 3136-3140.

DICKMAN, P.S., TSOKOS, M. & TRICHE, T.J. (1991). Biology of rhabdomyosarcoma: cell culture, xenografts, and animal models. In *Rhabdomyosarcoma and Related Tumors in Children and Adolescents*. Maurer, H.M., Ruymann, F.B., Pochedly, C. (eds), CRC Press: Boca Raton, Florida, USA, pp. 49-88.

DOLCETTI, R., DE RE, V., VIEL, A., PISTELLO, M., TAVIAN, M. & BOIOCCHI, M. (1988). Nuclear oncogene amplification or rearrangement is not involved in human colorectal malignancies. *Eur. J. Cancer Clin. Oncol.*, **24**, 1321-1328.

DYM, H. & YAFFE, D. (1979). Expression of creatine kinase isoenzymes in myogenic cell lines. *Develop. Biol.*, **68**, 592-599.

FLORINI, J.R. (1987). Hormonal control of muscle growth. *Muscle Nerve*, **10**, 577-598.

FLORINI, J.R. & MAGRI, K.A. (1989). Effects of growth factors on myogenic differentiation. *Am. J. Physiol.*, **256**, C701-C711.

FLORINI, J.R., ROBERTS, A.B., EWTON, D.Z., FALEN, S.L., FLANDERS, K.C. & SPORN, M.B. (1986). Transforming growth factor-beta. A very potent inhibitor of myoblast differentiation, identical to the differentiation inhibitor secreted by Buffalo rat liver cells. *J. Biol. Chem.*, **261**, 16509-16513.

FRANCHINI, G., WONG-STAAL, F., BALUDA, M.A., LENGEL, C. & TRONICK, S.R. (1983). Structural organization and expression of human DNA sequences related to the transforming gene of avian myeloblastosis virus. *Proc. Natl Acad. Sci. USA*, **80**, 7385-7389.

GABBERT, H.E., GERHARZ, C.-D., BIESALSKI, H.-K., ENGERS, R. & LULEY, C. (1988). Terminal differentiation and growth inhibition of a rat rhabdomyosarcoma cell line (BA-HAN-1C) *in vitro* after exposure to retinoic acid. *Cancer Res.*, **48**, 5264-5269.

GABBERT, H.E., GERHARZ, C.-D., RAMP, U., HOFFMANN, J., OSTER, O., OESCH, F. & DOEHMER, J. (1990). Enhanced expression of the proto-oncogenes fos and raf in the rhabdomyosarcoma cell line BA-HAN-1C after differentiation induction with retinoic acid and N-methylformamide. *Int. J. Cancer*, **45**, 724-730.

GARVIN, A.J., STANLEY, W.S., BENNETT, D.D., SULLIVAN, J.L. & SENS, D.A. (1986). The *in vitro* growth, heterotransplantation, and differentiation of a human rhabdomyosarcoma cell line. *Am. J. Pathol.*, **125**, 208-217.

GERHARZ, C.D., GABBERT, H.E., BIESALSKI, H.K., ENGERS, R. & LULEY, C. (1989*a*). Fetal calf serum and retinoic acid affect proliferation and terminal differentiation of a rat rhabdomyosarcoma cell line (BA-HAN-1C). *Br. J. Cancer*, **59**, 61-67.

GERHARZ, C.D., GABBERT, H.E., ENGERS, R., RAMP, U., MAYER, H. & LULEY, C. (1989*b*). Heterogenous response to differentiation induction with different polar compounds in clonal rat rhabdomyosarcoma cell line BA-HAN-1C. *Br. J. Cancer*, **60**, 578-594.

GUERRIERO, V. Jr & FLORINI, J.R. (1980). Dexamethasone effects on myoblast proliferation and differentiation. *Endocrinology*, **106**, 1198-1202.

HAMILTON, B.J. & DEFRANCO, D. (1989). Glucocorticoid and cAMP induction mechanisms are differentially affected by the p85gag-mos oncoprotein. *Proc. Natl Acad. Sci. USA*, **86**, 597-601.

KAPLAN, I., BLAKELY, B.T., PAVLATH, G.K., TRAVIS, M. & BLAU, H.M. (1990). Steroids induce acetylcholine receptors on cultured human muscle: implications for myasthenia gravis. *Proc. Natl Acad. Sci. USA*, **87**, 8100-8104.

LEIBOVITCH, M.-P., LEIBOVITCH, S.A., HILLION, J., GUILLIER, M., SCHMITZ, A. & HAREL, J. (1987). Possible role of c-fos, c-N-ras and c-mos proto-oncogenes in muscular development. *Exp. Cell Res.*, **170**, 80-92.

LIM, R.W. & HAUSCHKA, S.D. (1984). Rapid decrease in epidermal growth factor-binding capacity accompanies the terminal differentiation of mouse myoblasts *in vitro*. *J. Cell Biol.*, **98**, 739-747.

LOLLINI, P.-L., DE GIOVANNI, C., DEL RE, B., LANDUZZI, L., NICOLETTI, G., PRODI, G., SCOTLANDI, K. & NANNI, P. (1989). Myogenic differentiation of human rhabdomyosarcoma cells induced *in vitro* by antineoplastic drugs. *Cancer Res.*, **49**, 3631-3636.

LOLLINI, P.-L., DE GIOVANNI, C., LANDUZZI, L., NICOLETTI, G., SCOTLANDI, K. & NANNI, P. (1991). Reduced metastatic ability of *in vitro* differentiated human rhabdomyosarcoma cells. *Invasion Metastasis*, **11**, 116-124.

MATIN, A., CHENG, K.-L., SUEN, T.-C. & HUNG, M.C. (1990). Effect of glucocorticoids on oncogene transformed NIH3T3 cells. *Oncogene*, **5**, 111-116.

MAX, S.R., THOMAS, J.W., BANNER, C., VITKOVIC, L., KONAGAYA, M. & KONAGAYA, Y. (1987). Glucocorticoid receptor-mediated induction of glutamine synthetase in skeletal muscle cells *in vitro*. *Endocrinology*, **120**, 1179-1183.

MILLER, S.C., ITO, H., BLAU, H.M. & TORTI, F.M. (1988). Tumor necrosis factor inhibits human myogenesis *in vitro*. *Mol. Cell Biol.*, **8**, 2295-2301.

MINER, J.H. & WOLD, B.J. (1991). c-myc inhibition of MyoD and myogenin-initiated myogenic differentiation. *Mol. Cell. Biol.*, **11**, 2842-2851.

NANNI, P., SCHIAFFINO, S., DE GIOVANNI, C., NICOLETTI, G., PRODI, G., DEL RE, B., EUSEBI, V., CECCARELLI, C., SAGGIN, L. & LOLLINI, P.-L. (1986). RMZ: a new cell line from a human alveolar rhabdomyosarcoma. *In vitro* expression of embryonic myosin. *Br. J. Cancer*, **54**, 1009-1014.

NICOLETTI, G., DE GIOVANNI, C., LANDUZZI, L., SIMONE, G., ROCCHI, P., NANNI, P. & LOLLINI, P.-L. (1992). Induction of myogenic differentiation in human rhabdomyosarcoma cells by ionizing radiation, N,N-dimethylformamide and their combination. *Br. J. Cancer*, **65**, 519-522.

OLSON, E.N., BRENNAN, T.J., CHAKRABORTY, T., CHENG, T.-C., CSERJESI, P., EDMONDSON, D., JAMES, G. & LI, L. (1991). Molecular control of myogenesis: antagonism between growth and differentiation. *Mol. Cell. Biochem.*, **104**, 7-13.

PONTE, P., GUNNING, P., BLAU, H. & KEDES, L. (1983). Human actin genes are single copy for alfa-skeletal and alfa-cardiac actin but multicopy for beta- and gamma-cytoskeletal genes: 3′ untranslated regions are isotype specific but are conserved in evolution. *Mol. Cell. Biol.*, **3**, 1783-1791.

POON, M., MEGYESI, J., GREEN, R.S., ZHANG, H., ROLLINS, B.J. SAFIRSTEIN, R. & TAUBMAN, M.B. (1991). *In vivo* and *in vitro* inhibition of JE gene expression by glucocorticoids. *J. Biol. Chem.*, **266**, 22375-22379.

ROBBINS, K.C., DEVARE, S.G. & AARONSON, S.A. (1981). Molecular cloning of integrated simian sarcoma virus: genome organization of infectious DNA clones. *Proc. Natl Acad. Sci. USA*, **78**, 2918-2922.

RYDER, K. & NATHANS, D. (1989). Induction of proto-oncogene c-jun by serum growth factors. *Proc. Natl Acad. Sci. USA*, **85**, 8464-8467.

SCHIAFFINO, S., GORZA, L., SARTORE, S., SAGGIN, L. & CARLI, M. (1986). Embryonic myosin heavy chain as a differentiation marker of developing human skeletal muscle and rhabdomyosarcoma. *Exp. Cell Res.*, **163**, 211–218.

SCHWAB, M., ALITALO, K., KLEMPMAUER, K.H., VARMUS, H.E., BISHOP, J.M. GILBERT, F., BRODEUR, G., GOLDSTEIN, M. & TRENT, J. (1983). Amplified DNA with limited homology to myc cellular oncogene is shared by human neuroblastoma cell lines and a neuroblastoma tumor. *Nature*, **305**, 245–248.

SKLAR, R.M. & BROWN, R.H. Jr. (1991). Methylprednisolone increases dystrophin levels by inhibiting myotube death during myogenesis of normal human muscle *in vitro*. *J. Neurol. Sci.*, **101**, 73–81.

SOUTHORN, B.G. & PALMER, R.M. (1990). Inhibitors of phospholipase A2 block the stimulation of protein synthesis by insulin in L6 myoblasts. *Biochem. J.*, **270**, 737–739.

ULLRICH, A., COUSSENS, L., HAYFLICK, J.S., DULL, T.J., GRAY, A., TAM, A.W., LEE, J., YARDEN, Y., LIBERMANN, T.A., SCHLESSINGER, J., DAWNWARD, J., MAYES, E.L.V., WHITTLES, N., WATERFIELD, M.D. & SEEBURG, P.H. (1984). Human epidermal growth factor receptor cDNA sequence and aberrant expression of the amplified gene in A431 epidermoid carcinoma cells. *Nature*, **309**, 418–425.

WATSON, R., OSKARSSON, M. & VANDE WOUDE, G.F. (1982). Human DNA sequence homologous to the transforming gene (mos) of Moloney murine sarcoma virus. *Proc. Natl Acad. Sci. USA*, **79**, 4078–4082.

WAXMAN, S., ROSSI, G.B. & TAKAKU, F. (1988). (eds) The status of differentiation therapy of cancer. Raven Press: New York, NY.

WHITSON, P.A., STUART, C.A., HULS, M.H., SAMS, C.F. & CINTRON, N.M. (1989). Dexamethasone effects on creatine kinase activity and insulin-like growth factor receptors in cultured muscle cells. *J. Cell. Physiol.*, **140**, 8–17.